# How to make complexity look simple? Conveying ecosystems restoration complexity for socio-economic research and public engagement

**DOI:** 10.1371/journal.pone.0181686

**Published:** 2017-07-28

**Authors:** Julia Martin-Ortega, Klaus Glenk, Anja Byg

**Affiliations:** 1 Sustainability Research Institute, School of Earth and Environment, University of Leeds. Leeds, United Kingdom; 2 Land Economy Unit, Scottish Rural College. Edinburgh, United Kingdom; 3 Social, Economical and Geographical Sciences Group, The James Hutton Institute. Aberdeen, United Kingdom; University of Vermont, UNITED STATES

## Abstract

Ecosystems degradation represents one of the major global challenges at the present time, threating people’s livelihoods and well-being worldwide. Ecosystem restoration therefore seems no longer an option, but an imperative. Restoration challenges are such that a dialogue has begun on the need to re-shape restoration as a science. A critical aspect of that reshaping process is the acceptance that restoration science and practice needs to be coupled with socio-economic research and public engagement. This inescapably means conveying complex ecosystem’s information in a way that is accessible to the wider public. In this paper we take up this challenge with the ultimate aim of contributing to making a step change in science’s contribution to ecosystems restoration practice. Using peatlands as a paradigmatically complex ecosystem, we put in place a transdisciplinary process to articulate a description of the processes and outcomes of restoration that can be understood widely by the public. We provide evidence of the usefulness of the process and tools in addressing four key challenges relevant to restoration of any complex ecosystem: (1) how to represent restoration outcomes; (2) how to establish a restoration reference; (3) how to cope with varying restoration time-lags and (4) how to define spatial units for restoration. This evidence includes the way the process resulted in the creation of materials that are now being used by restoration practitioners for communication with the public and in other research contexts. Our main contribution is of an epistemological nature: while ecosystem services-based approaches have enhanced the integration of academic disciplines and non-specialist knowledge, this has so far only followed one direction (from the biophysical underpinning *to* the description of ecosystem services and their appreciation by the public). We propose that it is the mix of approaches and epistemological directions (including *from* the public to the biophysical parameters) what will make a definitive contribution to restoration practice.

## Introduction

The Earth’s ecosystems continue to be degraded at a pace that critically compromises their capacity to deliver services that are essential for human livelihood and well-being worldwide [1, 2, 3]. Ecological restoration is now a consolidated major strategy for redressing this threat [4, 5, 6] and is having an increasingly central role in global and regional environmental policy, e.g. [2, 3, 7, 8]. Consequently, restoration actions are increasingly being implemented throughout the world [9, 3].

Knowledge on the relationships between humans and nature in complex social-ecological systems is critical to the design of strategies to halt or reverse declining trends in ecosystem services provision [1, 10, 11, 12]. Therefore, for ecosystems restoration to be successful, restoration science [6] and practice needs to be coupled with an understanding of its multiple socio-economic aspects, from the drivers of degradation to the social costs and benefits of restoration outcomes [5, 13]. Moreover, for restoration to be successful it also needs to be socially desirable. In this sense, it is becoming widely accepted that a greater level of public engagement in environmental management in general and ecosystems restoration in particular is needed, both as a driver for policy-making and to support restoration action on the ground [14, 15, 16], as well as to identify and manage the conflicts that might emerge between conservation targets and public preferences [17]. This is coherent with a growing trend towards greater stakeholder participation in relation to adaptive management as emphasized by the Ecosystem Approach and post-normal science more generally [18].

Undertaking socio-economic research and public engagement to support ecosystem restoration requires conveying complex information in a way that is accessible for a wider public beyond specific academic audiences. How to do so in a way that the representation of ecosystems remains scientifically rigorous is a critical challenge [19, 20]. In this paper, we take up this challenge to support a stronger development of more effective ecosystems restoration at a moment in which restoration science is reshaping [5]. We use peatlands as a paradigmatically complex ecosystem [21] and Scotland as a case study to develop a transdisciplinary process to articulate a description of the processes and outcomes of ecosystem restoration. The aim is to support the development of tools that can be used for raising awareness to support policy making, as well as be used for social science research, for example concerning the assessment of public perceptions and preferences on restoration. This should also help in a better realignment of science and practice with regards to credibility, salience and legitimacy [22]. The ultimate goal is making a step change in the contribution of science to restoration practice.

It should be noted that our goal is to make ecosystem restoration complexity *look* simple, and not pretend that it can *be* simple. Also, while we present the tool that we finally produced in our case study for Scotland, our focus is to reflect on the process that led us to this tool in a broader sense regarding ecosystems restoration, beyond Scotland and peatlands.

## Peatlands ecosystems and their restoration

Peatlands cover over 400 million hectares of the Earth’s surface, i.e. over 3% [23]. They store a third of the world’s soil carbon [24], which makes them the largest and the most space-effective carbon store of all terrestrial ecosystems [25]. Climate change and land use and management, primarily agriculture and forestry following land drainage, is modifying the structure and function of these systems, potentially changing the global peatland greenhouse gas balance to a carbon source [26]. This threatens stocks of natural capital that have formed over millennia, undermining the adaptive capacity of peatland systems to climatic and other future changes [27]. Peatland degradation also compromises the delivery of other ecosystem services provided by peatlands, such as erosion control [28], water quality [29] and biodiversity [30].

These issues have increasingly become a focus with policy makers internationally. Peatlands are included in the Aichi Targets of the UN Convention on Biological Diversity (CBD) and can be accounted for in national climate change mitigation targets under the UN Framework Convention on Climate Change (UNFCCC) [31] restoration programmes are progressively being deployed across the globe [3].

Increasingly, these international policies and their national derivatives recognize the need for public support. They often set public awareness raising as a key objective for the success of these policies. For example, Scotland's National Peatland Plan published in 2015 to support Scottish Natural Heritage’s (SNH) Peatland ACTION, a multi-million peatland restoration programme deployed since 2012 (see next), emphasises the multiple benefits of peatlands to society and highlights that the future of peatlands should not only be of interest to the scientific community, policy makers, developers and land managers, but also tothe wider public. According to the Peatland ACTION, peatlands should be “no longer seen just as special interest habitats” and it also points out that peatland restoration can offer opportunities for community involvement, education and awareness raising [32]. This process needs to be facilitated by an improved understanding of perceptions held by the wider public regarding peatlands and their views and preferences regarding restoration [33], all of which are matters of social science research.

### Peatland status and restoration agenda in Scotland

Around 9–15% of Europe’s peatland areas are found in the UK of which more than 77% are located in Scotland [34, 35]. Peatlands cover more than 20% of Scotland’s land surface. Scottish peatlands mainly consist of blanket bog which is a globally rare habitat type [35].

In the past, peatlands in Scotland were mainly seen as either a source of peat or as wastelands to be converted to other productive uses such as forestry or agriculture [36, 33]. As a consequence, a large share of Scottish peatlands have been degraded to some extent leading to habitat degradation, release of greenhouse gases and problems with soil erosion and water regulation [37, 34]. More than two thirds of Scottish peatlands are damaged or degraded to some degree, and degradation is projected to continue if no action is taken [34].

In line with international trends, concerns around this damage led to the development in 2012 of Peatland ACTION, a programme by which the Scottish Government allocated Scottish Natural Heritage (SNH) £5 million to deliver: “restoration and management of peatlands to maintain carbon storage and encourage carbon sequestration to restore peatland ecosystem functions; enhanced ecosystem resilience to climate change; and to build peatland restoration capacity and understanding amongst land managers, contractors, advisors and the public”. Between 2013 and 2014, restoration activity has taken place in 5,580 hectares, while additional £3 million announced in June 2015 is expected to enable activity across another 3,000 hectares [32].

## Methods

There is a gap between the typical outputs of ecological process-based models which demonstrate changes in ecological parameters and the representation of such changes in terms that the general public can perceive [19]. This translation is the cornerstone of ecosystem services-based approaches to inform environmental decision making, in which some form of interplay between ecosystem services and human-wellbeing is addressed [38, 20].

To address this gap, we embarked in a transdisciplinary process involving input from three knowledge strands: natural sciences, social sciences and (peatland) management practice. A transdisciplinary approach is based on the principle that the integration of other actors in the knowledge production process, in addition to specialist academic knowledge, results in a ‘final knowledge’ that is anticipated to be greater than the sum of disciplinary components [39, 40, 41]. Transdisciplinarity is now advocated widely in social-ecological research [15] and in ecosystem restoration more specifically [5]. The principle is that the complex and dynamic nature of environmental problems requires flexible decision-making, embracing a diversity of ‘knowledges’ and values [14].

In this section we first describe the overall transdisciplinary process. Then we describe the protocols involved in collecting and analysing the data/information used to identify the key challenges to translate restoration complexity and to evaluate the usefulness of the process and its outputs. The research involved human participants in focus groups and a public survey (non-clinical) and received ethical approval from The James Hutton Institute and Scotland's Rural College.

### A transdisciplinary process to translate restoration complexity

The research process we applied was designed as consultative, rather than participatory transdisciplinarity, as defined by [41], i.e. having actors from outside academia responding and reacting to the research conducted. We also acknowledge that there are other interpretations of transdisciplinary research, where the emphasis is not on the inclusion of non-academic actors in the knowledge production process, but on the transcendence of the integration between academic disciplines. While led by social scientists, we believe our process also responds to this other interpretation of transdisciplinarity, since the representatives of the different disciplines were “encouraged to transcend their separate conceptual, theoretical and methodological orientations in order to develop a shared approach to the research, building on a common conceptual framework” [42].

Table 1 shows the stages of this process and the different knowledge strands engaged at each stage and Table 2 presents a summary of the organizations involved in this process.

**Table 1:** Stages of the transdisciplinary process.

Stage^a^	Aims	Strands of interaction^b^	Format of interaction
1	Understand the current knowledge base of peatlands processes, functions and ecosystem services delivery Identification of the policy agenda	Natural scientists; Practitioners and policy-makers	Workshops
2	Define the potential challenges associated with understanding peatland restoration and its public perceptions	Natural scientists	Bilateral dialogue
3	Development of a tool for conveying simplified restoration information	Natural scientists; Practitioners and policy-makers	Bilateral conversations; Policy makers and practitioners’ workshop
4	Testing and refining the tool with the public	Public	Focus groups; Survey
5	Validation and uptake	Natural scientists; Practitioners and policy-makers Public	Experts’ focus groups; Bilateral dialogue; Policy events; Learning module and condition assessment support tool

^a^Although stages are somewhat consecutive, there was some level of overlap between them and some of the tasks were interspersed (e.g. focus groups with the public also took place as part of stage 3 when developing the tool).

^b^The process is presented from the perspective of the social scientists leading this process, and therefore should be read as ‘strand of knowledge with which the social science strand interacts’.

**Table 2:** Main organizations involved in the transdisciplinary process^a^.

Name	Type of organization
The James Hutton Institute	Research (including hydrologists, ecologists, soil scientists, economists, environmental social scientists
Scotland’s Rural College (SRUC)	Research (economists)
University of Leeds	Research (including hydrologists, wetland and peatland scientist, economists).
University of Birmingham	Research (social and natural sciences)
Centre for Ecology and Hydrology	Research (including water ecologists, hydrologists and peatland scientists)
Scottish Natural Heritage (SNH)	Practice (peatland restoration practitioners)
Scottish Government	Policy-making (environmental and strategic research managers)
International Union for the Conservation of Nature (IUCN)–UK National Committee	Practice (nature conservation)
Scottish Environmental Protection Agency (SEPA)	Policy-making (environmental management)
ClimateXChange	Science-practice interface (climate change)

^a^Not all these organizations were involved with the same level of intensity. The greatest interaction was between representatives of The James Hutton Institute, Scotland’s Rural College, University of Leeds, Scottish Natural Heritage and the Scottish Government, who were involved in all stages of the process. The rest of the organizations took part at one or several stages of the ones presented in Table 1. This process was part of a broad research programme, mostly driven and taking place under the Scottish Government Rural Affairs and the Environment Portfolio Strategic Research Programme (2011–2016), and complemented by a series of additional research projects. For example, stage 1 took place in the context of the Valuing Nature Network Project ‘Valuing Peatlands’ funded by the UK’s Natural Environment Research Council (NERC) (2011–2012), and stages 2 and 3 involved scientists and funding from the Scottish ClimateXChange peatlands programme (2013–2014). Workshops with policy makers and practitioners in stage 3 took place in the context of the Scottish Government’s Ecosystem Approach Working Group (EAWG).

### Data gathering

#### Identification of challenges of ecosystem restoration

All the materials produced during stages 1 to 3 of the transdisciplinary process **(**Table 1) were recorded and stored systematically in chronological order during the process. These included workshops and policy events presentations, minutes and note-taking materials (e.g. flipcharts and post-it notes), as well as emails exchanged and minutes from bilateral conversations with natural scientists and restoration practitioners. At the end of the process, this material was all placed in one large analytical matrix which was then used to apply a grounded approach [43, 44]. This involved scrutinising the material several times to identify recurrent themes or topics which emerged from the data themselves rather than on the basis of pre-defined topics. All the parts of a text or other documents related to a particular theme or ‘code’ were then marked as such. In subsequent rounds of analysis of the material, codes were refined further, for example by identifying sub-themes within existing themes or codes.

#### Validation and assessment of usefulness

To assess whether we had succeeded in conveying complex information on ecosystem restoration in a way that can be used for public engagement while being scientifically rigorous, we set out to answer three questions: 1) can the public understand and relate to the information provided?; 2) do peatland specialists find the tool accurate enough?; and 3) is the tool useful for peatland management and policy-making?.

Data to answer the first question was collected during stage 4 (Table 1) using focus groups and a public survey Three focus groups with members of the general public were conducted in two locations in Scotland: one on the Isle of Lewis and two in the city of Aberdeen. The two locations were chosen due to their contrasting characteristics in relation to peatlands and the different relationships and experiences that we assumed people in these two areas would have with peatlands. The Isle of Lewis constitutes the northern part of the Outer Hebrides, off the west-coast of Scotland, and consists to a large extent of blanket bogs. It was chosen as a rural area where peatlands are still being actively used for domestic extraction of peat and grazing, although these uses are less widespread nowadays than in the past. Aberdeen is located on the east coast of Scotland, and was chosen as an urban, non-peat area where most people have limited personal experiences with peatlands.

Each focus group which lasted around 3 hours was advertised locally using social media, posters in public places and word of mouth. Participants were provided with a small monetary incentive presented as compensation for travelling. The focus groups in Aberdeen were held in October and November 2014, while the focus group in Lewis was held in July 2015.

In Aberdeen, 23 participants took part in the first focus group (9 male, 14 female, ages ranging from early 20’s to around 70), and 21 of these (8 male, 13 female) also took part in the second focus group. They came from a variety of professional and personal backgrounds, and apart from two people, did not have any direct experience of using peatlands (other than as the setting for recreational activities such as hill walking) or living in peatland areas. In Lewis, the focus group was attended by 14 participants (6 male, 8 female, ages ranging from around 30 to 70). Participants represented a mix of different backgrounds, including 3 crofters but also several people who were not native to Lewis. The main purpose of qualitative research as applied here is not to arrive at generalizations but to understand meanings in their context [45], and hence these groups were not meant to be representative of Scotland’s population. However, the participants in both areas included a wide spectrum in terms of gender, age and socio-economic background, and reported varying reasons for wanting to attend the focus groups (from a general interest in the environment and outdoor recreation to being offered some food at the workshop or “having nothing better to do that day”, etc.).

The focus groups were organized using a combination of break-out groups, plenary sessions and carousel activities, so that every participant had sufficient opportunity to express his/her views and interact with larger and smaller sections of the overall group. The main topics covered in the focus groups were: associations, experiences and memories related to peatlands; uses, activities and ‘good things’ associated with peatlands; conflicts and negative or ‘bad things’ associated with peatlands and peatlands’ degradation, restoration and management.

Four expert facilitators managed the focus groups, allowing for three individually managed break-out groups, with an additional facilitator monitoring time, participation and other logistical aspects. During discussions, notes were taken on a flip chart placed so that participants could see what was written down and allowing clarification of any mistakes or misrepresentations.

The materials produced during the focus group and notes taken by facilitators were transcribed and entered into qualitative data analysis software (Nvivo). The documents were coded using a grounded approach [43, 44].

The public survey used a questionnaire structured around the following issues:

- Information on peatlands, ecological condition, restoration and associated benefits
- Reasons for supporting (or not) restoration
- Views on where to restore (spatial prioritization of peatland restoration)
- People’s values for benefits resulting from peatland restoration expressed in monetary terms, elicited using choice experiments [46].
- Perceptions of peatlands including links to cultural identity.
- General attitudes towards the environment measured through the New Ecological Paradigm scale [47].
- Tool validation (see details next).
- Socio-demographic information about the respondents.

Of particular interest to the research presented here is the tool validation question in which respondents were asked in a Likert scale their level of agreement/disagreement with the following statements:

- The three ecological conditions look the same to me
- I now feel well informed about the different conditions peatlands can be in
- I can see clear differences between the three ecological conditions
- I didn’t understand what the three ecological conditions mean
- I would have recognized the differences in the ecological condition of peatlands in a real landscape without the descriptions that have been shown to me here

As well as the open-ended question at the end of the questionnaire in which respondents were invited to leave any comments, with the following questions included as prompts:

- Did you understand all content?
- Did you have difficulties in answering questions?
- Is there anything else you want to tell us?

Survey data include responses of 1795 Scottish citizens (aged 18 years old or older) conducted in February/March 2016. The survey was administered by a commercial marketing company. Respondents were recruited using the company’s family of panels comprised of 6,000,000 panellists across the UK (residents from out-with Scotland were disqualified at the start of the survey). The Panels have been recruited from a wide variety of Internet sites and through partnerships with leading brands to avoid the bias associated with limited source recruitment. The panellists were incentivised for their participation in the survey to help ensure reliable levels of response and that time was given for considered responses. Panel members were pre-screened and invited to take part in the survey, thereby avoiding people who have completed surveys in the last months. Response times were monitored and ‘speeders’ (i.e. those taking the survey in 10 minutes or less) were disqualified.

A randomized quota based approach was used to sample from the online panel with age and gender as ‘hard’ quotas and a ‘soft’ quota for social grade. Randomization and quota filling process was monitored through a bespoke programming scheme put in place by the web-programming team of the University of Leeds.

Raw data from the survey can be found in Supporting Information S1 Dataset. Table 3 displays the key socio-demographic characteristic of the sample and of Scotland’s population. We consider the sample to be fairly representative of the Scottish population and appropriate for our purposes. It would seem that higher education levels are over-represented; however, it should be noted that Scotland’s census measures education level for the population of 16 years old and above, while our sample includes population of 18 years old or older, which partly explains Level 0 under-representation. Moreover, the 2.3% of respondents who “prefer not to tell” their educational attainment are likely to fall within the Level 0 group. Supervisory/clerical/junior level is over-represented while the skilled manual level is under-represented. Similarly to above, “prefer not to tell” responses are likely to correspond to un-skilled workers, suggesting that that segment is well represented. The sample displays higher average household income, however it should be noted that there is about 65% of non-responses to the income question.

**Table 3:** Key socio-demographic characteristics.

Variable	Sample	Overall Population (Scotland)
Gender distribution		
Female	50.10%	51%
Male	49.90%	49%
Age distribution (years old) *		
18–24	8.0%	11.9%
25–44	36.8%	33.0%
45–64	34.2%	34.2%
≥ 65	21.0%	20.9%
Yearly household income		
GBP per year	£40,155	£38,337
Educational attainment (highest achieved Scotland census level)		
Level 0	12.3%	26.8%
Level 1	18.1%	23.1%
Level 2	18.5%	14.3%
Level 3 and above	48.1%	36.0%
Prefer not to tell	2.3%	-
Social grade (employment-based)		
Higher and intermediate	22.1%	19.0%
Supervisory, clerical, junior	40.1%	32.0%
Skilled manual	9.7%	22.0%
Semi-skilled, un-skilled	18.9%	28.0%
Prefer not to tell	9.1%	-
Average household size		
Persons per household	2.34	2.25
Urban/Rural population		
Urban	66.7%	69.9%
Rural	33.3%	30.4%

Sample’s figures are based on 1795 respondents, expect income (629), educational attainment (1793) and employment levels (1791). Source of population data: Scotland Census (2011) by National Records of Scotland (http://www.scotlandscensus.gov.uk/).

^*^Population figures include population ≥16 years old. Survey sample includes respondents ≥18 years old.

The aspects of the survey reported in this manuscript have been analysed using descriptive statistics (frequencies) of answers to close-ended questions. Open-ended answers were analysed using a categorical coding system differentiating: positive/supportive, negative/dismissive statements about the information presented on the survey, statements with respect to general aspects of land/environmental management.

Validation from peatland specialists and peatland managers and policy makers was obtained during an expert’s focus group (stage 5, Table 1). They were first presented with the tool and were directly asked if they found it rigorous enough and acceptable within their area of expertise/for their work. Subsequently, the tool was used as boundary object in a series of exercises aimed at reaching consensus amongst the specialists in areas where it had been previously difficult to reach that consensus (e.g. extent of peatland area in Scotland and its ecological condition and predictions on future state of peatland with and without restoration). Usefulness of the tool for this specialist audience was assessed in terms of its instrumentality in achieving such consensus. Uptake of the tool from natural scientists and restoration practitioners was monitored through subsequent direct communication.

## Complexity challenges of ecosystem restoration

We identified four challenges that apply to ecosystems restoration generally and that we describe next. After that, we explain how we addressed the challenges so that a tool for communication and elicitation of public views and preferences with regards to restoration could be developed and used in policy-making.

### Restoration outcomes

Ecological restoration involves assisting the recovery of an ecosystem that has been degraded, damaged or destroyed, typically as a result of human activities, so that its characteristics prior to degradation can be re-established [48, 9]. From the public’s perspective, it could be argued that what matters is people’s perception of the *outcomes* of restoration in relation to degradation, i.e. what are the services and their level of provision that the restored environment provides to people and that are recognised as such (e.g. can the ecosystem be enjoyed recreationally? does the ecosystem host unique species that people care about?, etc.). However, interacting with natural scientists it soon becomes clear that it is not possible to isolate the outcomes of restoration from the specific functions and processes involved in particular instances of degradation and restoration. Basically, the outcome of restoration always ‘depends on something’.

The relationship between ecosystem functions and processes and ecosystem service delivery is very complex [19]. For example, in the case of peatlands, which are the result of organic matter accumulation in the ground over millennia, carbon is stably stored in deep water-logged layers, but depending on the relative magnitude of fluxes of two different forms of carbon (CO_2_ and CH_4_), peat formation might either have a net cooling or warming effect on the climate [21]. Also, in rain-fed blanket bogs, peat can retain and immobilize polluting substances acting as a water quality regulator but, at the same time, they are natural sources of dissolved and particulate organic carbon that colours the water making it unsuitable for drinking and having some negative effects on downstream fauna [49, 50]. When pressure-response functions are then brought into the picture, it gets even more complicated. Each anthropogenic pressure (e.g. drainage or managed burning of peatlands) may impact on multiple ecosystems and each ecosystem function may be affected by multiple anthropogenic pressures, leading ecosystem services to different ‘impact pathways’ [21]. Moreover, different ecosystem services might display contrasting trajectories during restoration, leading to conflicts and trade-offs [4]. All this is ecosystem-specific, since the functioning and service delivery role of ecosystems can be very different between, for example, rain-fed bogs and ground and surface-water fed wetlands [21], and in many instances, even site-specific (e.g. in relation to the ‘original’ carbon content of the soil and geomorphological characteristics such as slope). Finally, restoration can mitigate certain anthropogenic pressures (e.g. through re-wetting and re-vegetation), but other global factors, such as climate change, will continue to exert pressure and have an effect on the trajectory of ecosystem services, leading to variable outcomes.

In essence, the outcomes of restoration are ultimately linked to the processes underpinning the functioning of the ecosystem, and to provide a more rigorous representation of the ecosystem services delivery associated with restoration, these need to be related to such processes.

### Restoration reference

It is accepted in the restoration literature that effectiveness of restoration projects is to be evaluated against a reference, which in practice means the attributes of an un-degraded ecosystem [4]. What that reference should be is debatable [5], i.e. either a presumed historic state or an agreed extent of natural or semi-naturalness. It is therefore difficult to decide what the goal of restoration should be.

This leads to two interrelated challenges: i) how to define and describe the status quo (i.e. what is the current state of the ecosystem and how might it look like in the future if no action is taken, recognizing the heterogeneous nature of the current ecological state across the landscape and conveying for the fact that *current* is different to *business as usual–*i.e. if no restoration takes place); and ii) how much restoration can be achieved (i.e. what will the ecosystem look like if restoration takes place), recognizing the differences in response to available restoration practices. In the case of peatlands, this involves determining their extent, i.e. where areas can be found that classify as peatlands, which is not at all straightforward as proven by the existing discussions and varied evidence in peat soil modelling [51]. Furthermore, information needs to be obtained on how much of the existing peatland is degraded in order to know how much can be potentially restored. Additionally, the degree of degradation needs to be known, since that also determines the potential for restoration. Heavily damaged peatlands not only have higher current levels of GHG emission, but they are also projected to emit more emissions in the future, therefore the potential emissions savings from restoration are higher in comparison to less degraded peat areas [52].

### Varying restoration time-lags

Some ecological responses are rapid, while others are subject to significant time lags over years or even decades [21]. These lags can be due to capacity factors (e.g. in the case of peatlands, the ability of soils to buffer acidification) or to decreased ecosystem resilience [21]. Therefore, there may be a delay in ecosystem recovery following the reduction or removal of an anthropogenic pressure. In some cases, restoration activity could even have a transient negative impact on other ecosystem functions, as has been observed in increased methane emissions following peat re-wetting [21].

Restoration time-lags, may vary across different ecosystem services. For example, GHG benefits might only arise after 20 or 25 years, while biodiversity and water quality impacts may be realized earlier, e.g. there are rapid water purification benefits that can be visible in less than 5 years [29]. They also vary across sites depending on the current state of the ecosystem. For example, the time required to achieve significant GHG emissions reductions from peatlands can vary from a few years in the case of less severely damaged peatlands to decades for more heavily damaged peatlands [52].

This makes it difficult to explain to the public what can be expected from restoration and when can it be expected.

### Spatial units for restoration

As explained, biophysical delivery of services from an ecosystem vary across different sites as a consequence of different environmental conditions and current level of degradation. Moreover, the placement of restoration activities within a certain broader area can also affect ecosystem service delivery [53]. In the most straightforward cases, changes in ecosystem service flows resulting from the restoration of one spatial unit are independent of any changes in surrounding units. In most cases, however, the assumption of spatial independency of ecosystem services flows does not hold true due to spatial interactions, connectivity between spatial units (e.g. upstream-downstream effects, spill-over effects) and non-linearity in ecological response with respect to the area under restoration [53]. For example, peatland restoration can result in reduced flood risk depending on environmental factors, such as the precipitation patterns across the catchment, but also on the placement of the restoration activity within the catchment [54, 55].

There is therefore a challenge in defining the spatial units in terms of the ecosystem services flows and impacts of restoration and how are these presented to the public. In some types of ecosystems, e.g. river systems, it might be easier to determine those spatial units using well established biophysical boundaries (e.g. hydrological catchment boundaries). However, for many other ecosystems including peatlands, those boundaries might be less clear cut, requiring the creation of ad-hoc criteria based on characteristics such as carbon content, soil bulk density, or existing vegetation patterns.

## How we addressed the challenges

### Representing restoration outcomes

As established earlier, to present a rigorous picture of what restoration can entail in terms of outcomes, these need to be linked to the processes that produce them. However, presenting the public with the full array of options and the conditions they depend on is simply not feasible. A way of overcoming this is to describe the provision of ecosystem services in relation to an ecosystem’s condition [4]. The description of the condition with a simple narrative describing key patterns of the processes and the outcomes should convey more complex information in a relatively simple manner. In accordance with natural scientists we selected a set of core characteristics (or attributes) that represent the key ecological parameters of those processes and the conditions that enable them, linking them to the ecosystem services (outcomes) that they provide.

Following this approach, we set out to establish discrete peatland ecological conditions, representing different ecological status with varying levels of degradation (from none or natural state to heavily degraded). We acknowledge that restoration does not necessarily happen as regime shifts and that there are gradients determined by specific response functions as explained earlier [5, 21], but we believe that if the discrete categories are accompanied with the appropriate narratives, they can allow a representation of processes that reflect the idea of progressive rather than discrete change.

**Fig 1:**
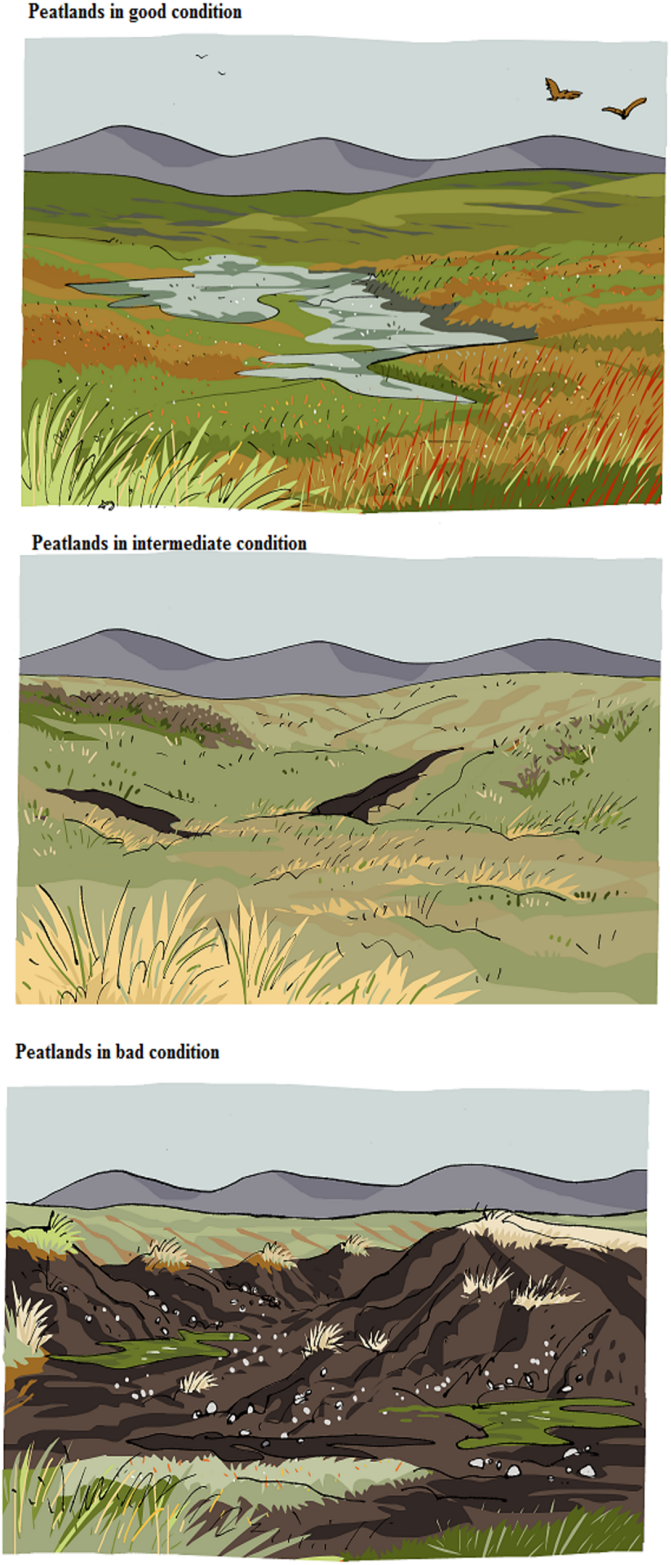
Categories of peatland condition. Own elaboration. The images were drawn by Ximena Maier.

Populating each of the peatlands conditions with the narratives describing the processes and the outcomes proved challenging. Initially, we tried to find pre-defined ecological categories that we could simplify. For example, we looked at the Common Standards Monitoring Guidance for Upland Habitats produced by the Joint Nature Conservation Initiative [56]. But our discussions with natural scientists about defining a limited number of simple but meaningful categories of peatland conditions usually led to vague ‘it depends’ kind of answers due to the many complexities outlined above. Eventually we tried an innovative exercise that we recommend for interdisciplinary work. Using SNH’s peatlands photographic archive in a workshop, we initiated a discussion in which we asked peatland specialists to qualify pictures of individual peatland sites as being in good, bad or intermediate condition (without having pre-defined what each of these meant). Whilst it had previously proven difficult to get the specialists to reduce the complexity of peatland conditions down to a limited number of categories and key characteristics, it proved much easier to start from photographs of existing peatlands (which were usually well-known to them) and derive a more general classification and defining characteristics from the photos. In this respect, the photos worked as *boundary objects* that enabled the discussion and consensus [57]. Once the photos had all been allocated to each of the groups, it became obvious that all the photos within one group shared a common set of features. For example, all the photos in the ‘bad ecological condition’ group included clear signs of erosion, with the bare rock visible and isolated ‘peatland stacks’, while in the peatland in the ‘good ecological condition’ group, the water table was visible displaying a red-green-brown mosaic colour pattern of small grasses, etc. This allowed us to establish three peatland conditions characterized by key processes leading to outputs in terms of carbon sequestration, support for wildlife and water quality regulation, which were easily recognisable to the public, reflected the reality of Scottish peatlands (all photos were taken at Scottish sites), and were generic enough to reflect the broad Scotland’s peatland landscape rather than individual sites. The peatland categories were then translated into three stylized drawings created by a graphic artist (who also used the photos as reference) and associated with narratives describing the processes and outcomes. Several iterations between the artist and the peatland specialists ensured that ecological rigour was maintained despite the simplification. The final result, which was further tested with the public and experts in respective focus groups (stages 4 and 5), is presented in Fig 1 (images) and Table 4 (narratives). The information and images are open access under the conditions of the Creative Commons copyright and can be freely (download here: http://www.see.leeds.ac.uk/peatland-modules/embeds/index.php)

**Table 4:** Narratives describing the peatland condition categories.

**Good condition**
In good condition, there is plenty of water, so it is visible on the surface, slowly flowing through larger and smaller pools. You will see small grasses and especially the peat moss that grows well in wet conditions. The moss stores lots of water and makes the peatland appear in a typical red-green-brown mosaic. Peatlands in good condition continue to grow by adding more and more layers of peat. While growing, carbon is taken up from the atmosphere as carbon dioxide (co2) and stored as peat. Water that flows from peatlands in good condition is usually clear and of good quality. This means less need for water treatment. The water quality is also good for fish living downstream, especially of salmon and trout. Peatlands in good condition are home to various birds and wildlife species. This includes waterfowl and wading birds such as curlew, and predators such as hen harrier and red kite.
**Intermediate condition**
In peatlands in intermediate condition, water has been taken off the land by creating channels for drainage. This allows activities such as livestock grazing. Surface water is rarely visible. With less water on the land, taller plants can grow, like cotton grass, or small bushes like heather. Peatlands in this condition are not very colourful. However, if heather grows in the area and is in bloom, its purple colour stands out. Signs of bare peat start to appear as dark patches. Sometimes peatland of intermediate condition is burned regularly, to create conditions for grouse shooting. This leaves characteristic patterns of burned and unburned land in the landscape. Peatlands in intermediate condition have stopped growing. No additional peat layers are added. Instead, peat layers gradually shrink, releasing a moderate amount of carbon to the atmosphere, where it contributes to climate change. Water flowing from such peatlands can be of lower quality. Water can be slightly murky, especially after a heavy rainfall. This can affect the fish population downstream, including salmon and trout, and increase the need for water treatment. Peatlands in intermediate condition may still harbour some of the wildlife that is present in peatlands in good condition. However, it is less abundant and some of the wildlife may not be found any more. It is also more likely that you will see managed species such as deer, sheep and grouse.
**Bad condition**
Peatlands in bad condition have been drained for a longer time. The forces of water and wind (erosion) have now exposed larger areas of bare peat. Deep gullies and drenches are formed. Rarely any plant grows on the areas that are exposed. Patches of grasses or heather are still found on ‘islands’ in between exposed bare peat. The exposed bare peat areas will continue to grow, leaving less plant cover as protection on the surface. Peat will continue to be lost until the solid rock surface emerges. Peatlands in bad condition lose carbon at a high rate. They have turned into a severe ‘source’ of carbon to the atmosphere, where it contributes to climate change. Water that flows downstream is of bad quality. It is often murky and can be dark brown from soil components in the water, especially after heavy rainfall events. The bad water quality will affect fish downstream. It is not suitable for human consumption and therefore needs a lot of treatment. Peatlands in this condition are home to little wildlife. Not many plant and animal species can be found.

**Fig 2:**
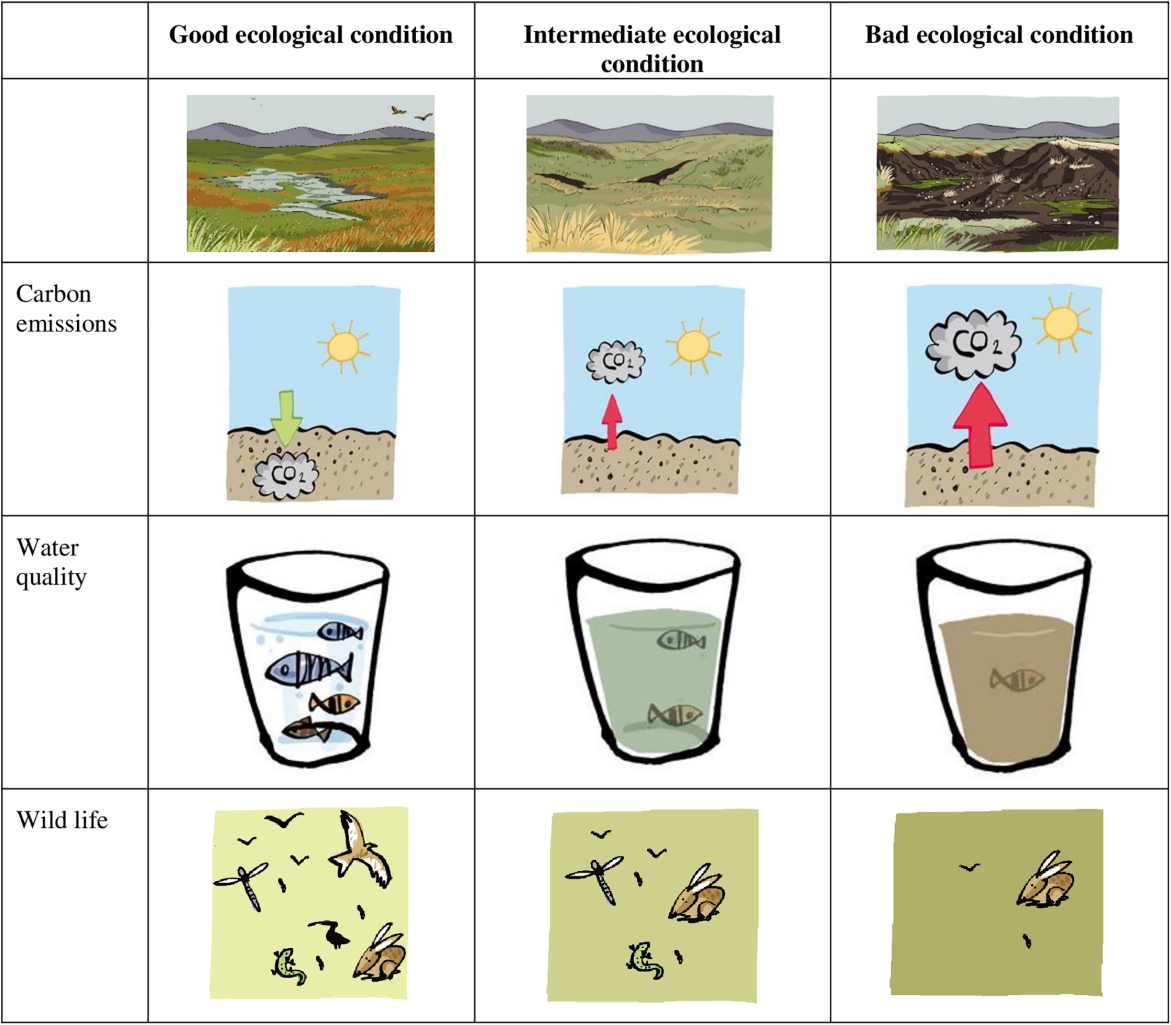
Iconic representation of restoration benefits in terms of carbon sequestration, water quality and wildlife habitat (accompanying the images in Fig 1 and the narratives in Table 4). Own elaboration. The images were drawn by Ximena Maier.

The three specific ecosystem services which experts often emphasise in relation to peatland (carbon sequestration, support for wildlife and water quality) were further depicted by icons also produced by the graphic artist in ways that could be related to the three different peatland states and their associated narratives (Fig 2). When presented to the public, the narratives also included reference trade-offs between peatland restoration and alternative land use changes. For example, the tool includes the following statement "Peatlands which are in intermediate condition can be used for livestock grazing and field sports (grouse), but if they deteriorate to poor condition all of these uses and activities are severely impaired”.

It should be noted that although the narratives are associated with varying degrees of provision of the three key ecosystem services, they do not include quantitative information, e.g. the bad ecological status reports on higher levels of carbon emissions than the intermediary level, but it does not specify how many units of carbon is released in a quantitative manner). Although some of the literature on ecosystem services argues that quantitative information on ecosystem services flows is required to undertake any analysis [21], others argue that the need for quantification depends on the purpose for which information is used [20]. Under certain circumstances, numbers can provide a false sense of accuracy and certainty that could be misleading, i.e. there is a danger of reducing complexity down to a single number that is then mistaken for reality [58].

### Establishing the restoration reference

We followed the recent trend in restoration ecology to frame restoration with a future rather than a historical focus [5]. During one of the experts’ focus group, it was agreed that a version of [51]’s map of soil carbon stocks for Scotland provided the appropriate reference base for current peatland cover area in Scotland. This allowed us to establish a percentage area of Scotland to be considered as peatland. The next exercise, also involving consensus in a focus group, was to allocate approximate percentage figures to each of the three ecological conditions that we had previously defined (i.e. how much of Scotland’s peatland area is currently in good, intermediate or bad condition).In addition, a carefully facilitated exercise using pre-established ‘guessed’ figures as *templates for knowledge exchange* [59] enabled the peatland scientists to agree on a target figure of potentially feasible restoration and future distribution of peatland status across the three categories. Because this latter exercise was particularly difficult for participants to predict as it would depend on the restoration effort and external drivers such as climate change, we asked them to think of a pessimistic and an optimistic restoration scenario, so that a lower and upper bound of restoration potential could be established.

### Coping with varying restoration time-lags

We explored the public’s sensitivity to various restoration time horizons (i.e. time by which restoration would be completed) and found that it did not make a different to people whether the time horizon for restoration was 15 or 30 years in this particular case. Focus groups participants stated that it might make a difference to them if the benefits from restoration were achieved in less than 15 years, for example, in 5 years, or on the contrary, in 100 years or more (i.e. their appreciation for restoration might increase if results can be obtained in the short-term and, opposite, their appreciation for restoration might decline if benefits can only be realized beyond their lifetime). To accommodate this and for the varying time lags (i.e. the fact that not all services might be delivered at the same time) and after consultation with the natural scientists, the communication tool referred to the fact that: *“Complete recovery might be achieved only after a few decades*, *but improvements in carbon emissions*, *water quality and wildlife will already be evident after 3 to 5 years*.*”*

### Defining units of restoration

**Fig 3:**
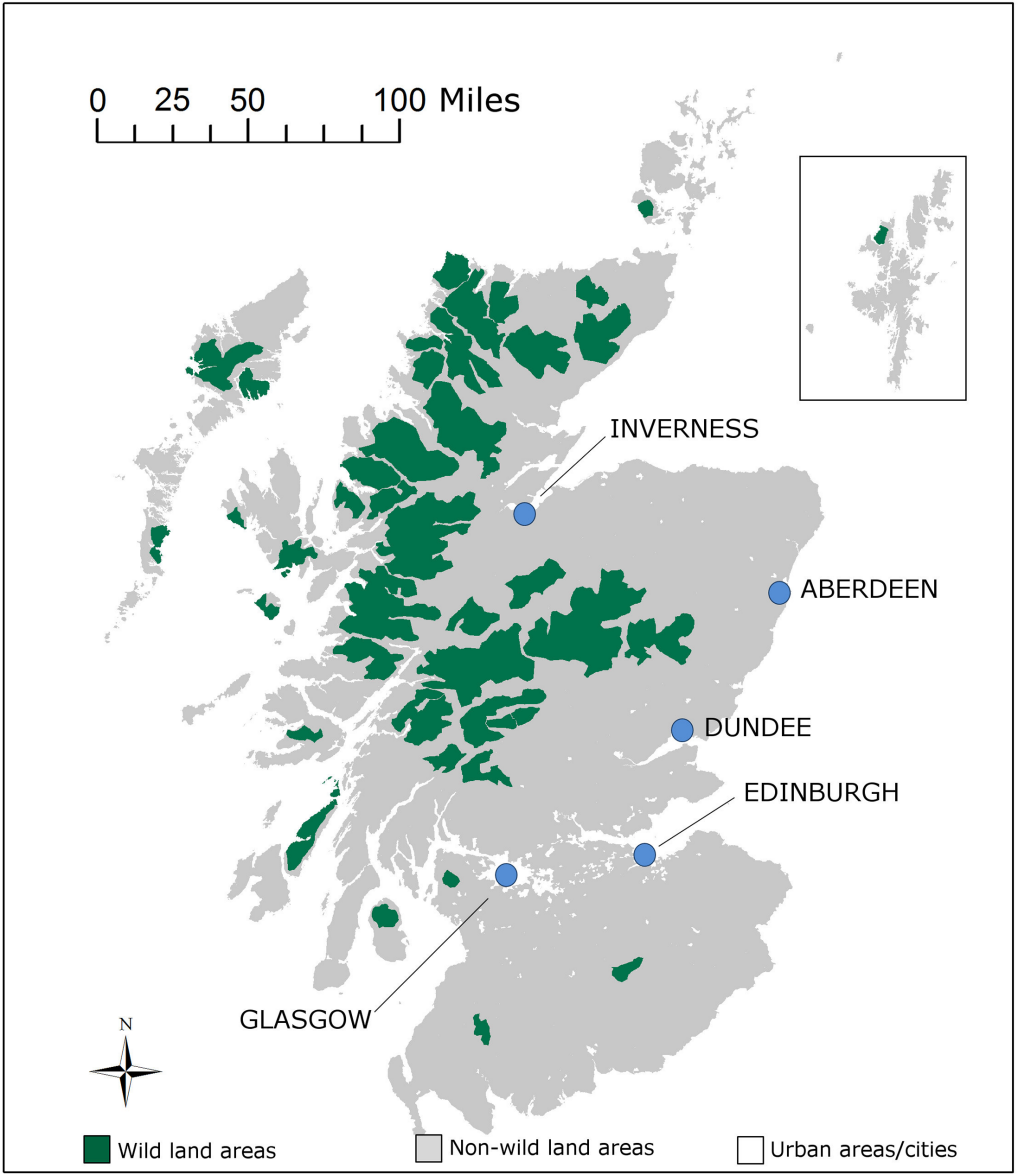
Map of peatlands in wild land areas. Own elaboration using SNH’s open access wild land areas map [61].

**Fig 4:**
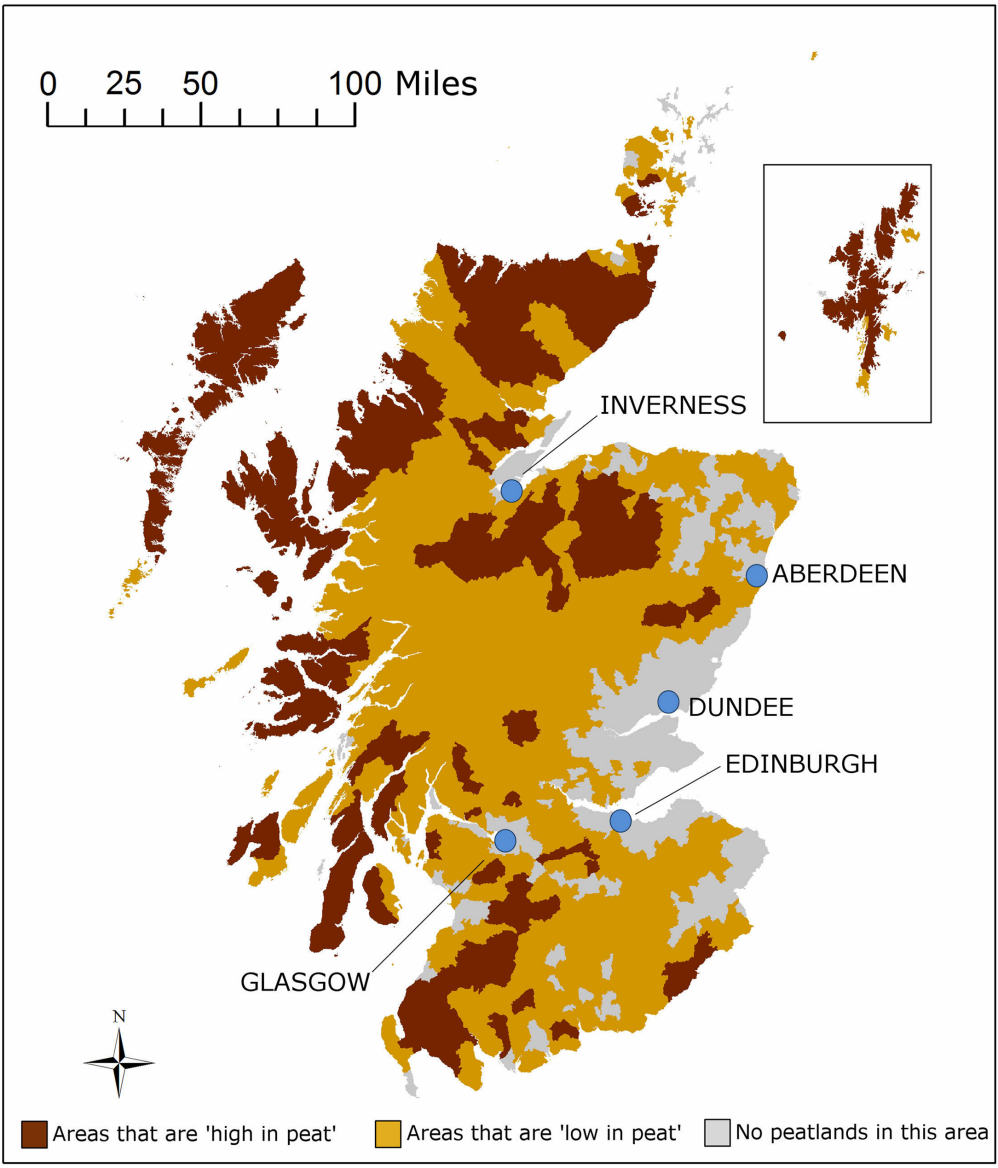
Map of high/low peat concentration. Own elaboration with the support of Matt Aitkenhead from The James Hutton Institute.

In a focus group we first identified the spatial criteria that, given the option to spatially prioritize restoration, are most relevant from the public’s perspective. Two spatial criteria emerged as most relevant for the public: i) level of peat concentration, i.e. areas with high or low concentration of peat (referred to by focus groups’ participants as *“the heart of it”* or “*what little is left”*) and ii) level of remoteness or accessibility (referred to as areas *“where restored peatlands can remain undisturbed”* versus *“accessible areas where they can be easily enjoyed”*). Once the spatial criteria that mattered to the public were identified, we set out to see if we could relate them back to meaningful spatial units from an ecosystem’s perspective. For that, we again sought the input of peatland specialists. For the first criteria (high or low peatland concentration) we co-constructed with a soil mapping specialist a ‘parish peat map’, in which parishes boundaries were used to define areas of high and low peatland concentration (more than 30% carbon content, less than 30% carbon content or none or insignificant carbon content). For the second criteria, we were advised to use Scotland’s wildland areas map [60], in which wildland areas are defined according to criteria of perceived naturalness, rugged or challenging terrain, remoteness from public mechanised access and lack of built modern artefacts [61]; a description that fitted well with what we had elicited from the public in the focus group. Resulting maps are presented in Fig 3 and Fig 4.

What is interesting from this process is that rather than trying to “force” relevant ecological spatial units into the tool and make the public “learn” them, we identified first what matters for the public in terms of spatial criteria and then we looked for what could be appropriate ecological parameters that could fit those definitions. This means that, for this particular aspect, we tested the possibility of inverting the conventional way of translating ecological complexity into something simple, and we started from what was simple enough for the public and worked our way back to the ecological parameters.

## How do we know what we did was useful?

### Do peatland scientists find the tool accurate enough?

The tool was essentially co-produced with natural scientists from the outset (see Table 1), but there was always a risk that the final product, which necessarily included numerous simplifications, might have been perceived as unsatisfactory by them. In the validating experts’ focus group (stage 5, Table 1), the peatland specialists confirmed that they were comfortable with the ecological conditions and the associated narratives and pictorial representations. Furthermore, as explained earlier, the three peatland categories were used in that focus group to elicit additional information from the experts which had not been possible to convey previously. Until that stage of the process, it had not been feasible to achieve a consensus amongst the scientists involved in the process as to how much peatland there is in Scotland at different levels of degradation. Having the peatland status categories as a framework of reference made it possible for them to achieve a consensus in this matter. For us, this is a good indication of the usefulness of the tool for natural scientists.

Further indication of the usefulness of the tool was its adoption by a different group of natural scientists who were not involved in the development process. They adopted it in an interdisciplinary research proposal aimed at understanding ecosystem stocks and tipping points in UK peatlands (submitted to the UK Natural Environment Research Council research call in May 2016). In the proposal, the tool was proposed as the reference framework for linking the outputs of a peatland process-based model, named DigiBog [62], to economic endpoints of degradation and restoration. The fact that this new set of natural scientists was willing to use our tool was a positive result. Even more, the research proposal was successful, the research project was funded and is ongoing at the time of writing.

### Can the public understand and relate to the information provided?

Focus group participants (stage 4, Table 1) demonstrated that they clearly understood the differences and characteristics of the three ecological statuses. Interestingly, when presented with the icons representing the benefits of restoration in terms of carbon sequestration, water quality and wildlife habitat, participants did not necessarily find them all very clear but were able to suggest alternative forms that were finally adopted. To us, this is again evidence of their usefulness. It enabled a meaningful discussion about what benefits and in which form are people can best relate to (contains the final versions of the icons after workshop’s modification).

**Fig 5:**
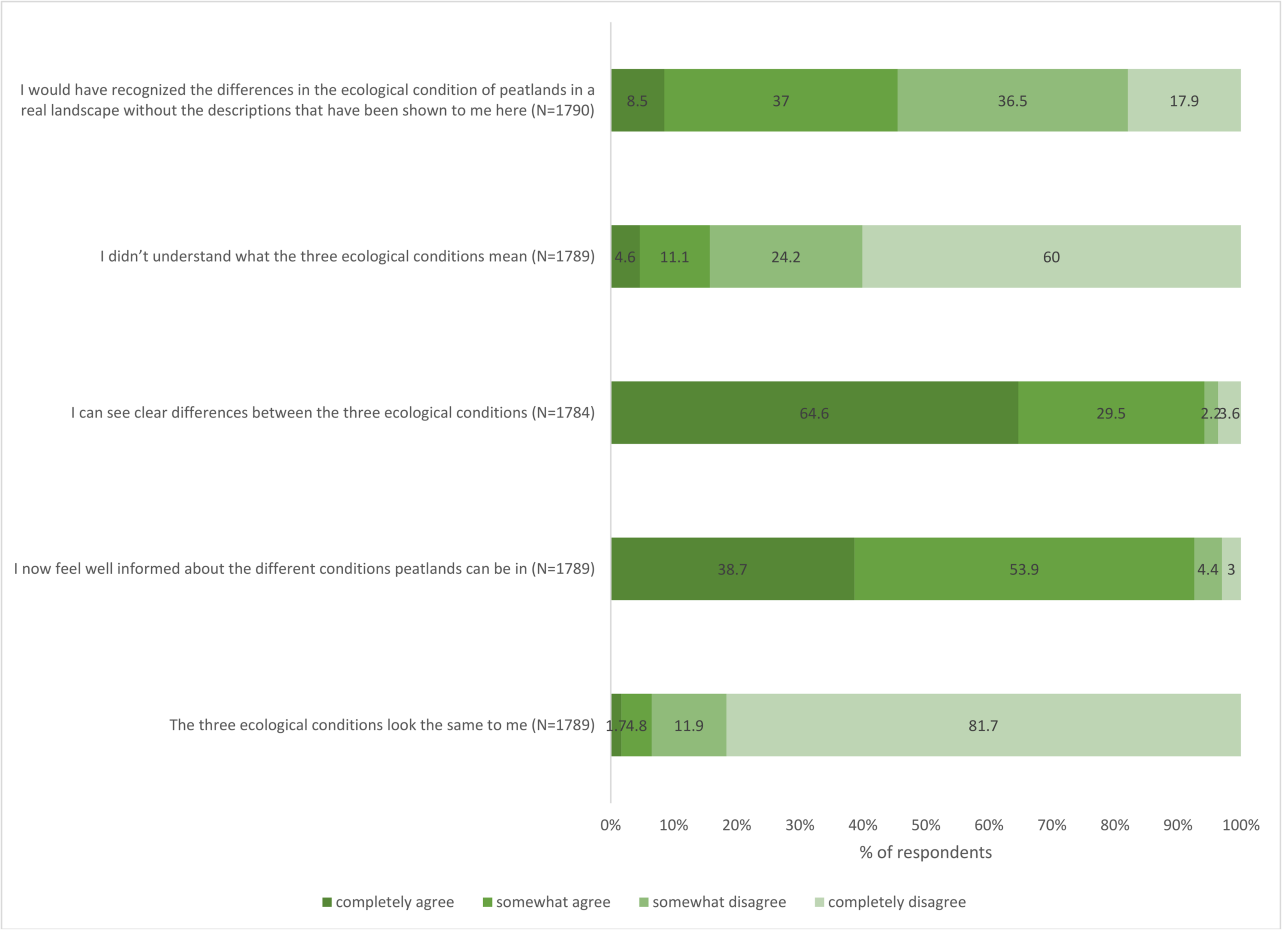
Survey responses to the usefulness and credibility of the peatland status categories, their associated benefits and describing narratives.

In the survey, respondents were explicitly asked their opinion on the three peatland categories. Fig 5 shows the results, which indicate clearly that the tool was understood and found credible.

At the end of the survey, respondents were invited to leave comments and provide feedback on the survey. About 17% of respondents did, which in our experience is a high number for a survey of this kind. Using a coding system, we are able to establish that about 65% of those comments were general positive comments on the survey. For illustration, Table 5 includes a selection of quotes of these positive comments, which provide evidence of the tool’s capacity of successfully engaging with the public in this complex topic. These comments reveal that many respondents found it informative and educational, and that it brought to their attention something that they had not thought about before. Several expressed their interest in what was presented to them being carried forward in policy or even engaging with it personally. There was also negative feedback, which amounted to 12% of the total of the comments. The negative comments regarded mostly a perception of a conservationist bias of the survey and only few comments indicated that respondents found the information somewhat overwhelming. A further 12.9% added comments on general environmental management issues, the role of the government, who should have responsibility over peatland restoration, etc.; which is again an indication of the capacity of the tool to enable a public discussion on this topic providing useful insights for policy-makers. The remaining 9.6% corresponds to specific comments on technical issues with filling in the survey.

**Table 5:** Selected quotes from survey respondent’s open feedback.

Positive feedback on the survey	Negative feedback on the survey	General comments about environmental management and related topics
*“Very interesting*. *did not realise the extent of the damage to the peatlands and the need to improve them”*	*“go hug trees”*	*“Apart from an obligation on landowners*, *extractors and business owners who have profited from peatlands should take responsibility“*
*“Glad to see this going ahead*. *Hopefully policy will follow”*	*“This survey was very biased toward conservationism*, *very little was said about the other economic or ecological impact of other use of the Peatland”*	*“Those people responsible for damaging peat bogs should have to pay to repair them*. *Peat bogs have been drained over the years and this now leads to flooding caused by excessive water runoff*. *Land owners who have drained their upland areas are partly to blame for flooding downstream*. *Peat bogs should be repaired but not at the cost of the tax payer who has never done anything to harm them directly”*
*“Very enjoyable and educational*. *Different than the norm*. *No problems whatsoever in understanding or progressing with the content*. *Look forward to hearing more on this matter”*	*“Would it have been better spending the money spent on surveys on dealing with the problems?”*	*“While we need to retain some peatland and restore some other we do not need simply to turn the clock back 1000 years*. *The money should be used where the most benefit will be gained—the principle of diminishing marginal returns will show when to stop”*
*“The survey highlighted and area which I had not really focused my attention to”*	*“Found all the initial information a little overwhelming and didn't really feel qualified enough to choose between the options with all the information”*	*“it would be interesting to know about the impact of the whisky industry on the peatlands in Scotland”*
*“This questionnaire was very educational*: *I've learned a lot about peatlands”*		
*“I found it extremely interesting as I knew nothing about peatlands before today”*		
*“the pictures and graphs were excellent are they available for use?”*		
*“I found this an interesting survey*, *I have never thought of the peatlands like this before and it has made me more aware of them and their different states*. *I regularly was up […] and can relate to the different peat states which were discussed in this survey*. *Thank you for this opportunity to learn”*		
*“This was one of the most informative surveys I have ever completed*. *It should go to everyone in the country*! *Thank you”*		
*“Do you have any jobs/voluntary work regarding Peatland conservation? If so email me at …@hotmail*.*co*.*uk”*		

### Is the tool useful for peatland management and policy-making?

As well with natural scientists, the development of the tool involved peatland restoration practitioners from the outset and was specifically developed to serve restoration decision-making purposes. Evidence of this success can be highlighted by the fact that the three peatland categories, the narratives and the associated description of benefits have been incorporated into the SNH communication strategy.

Specifically, the tool has been adopted as:

- A *learning module* to be included in SNH’s general public awareness raising strategy (the learning module is available here: http://www.see.leeds.ac.uk/peatland-modules/?type=learning).
- An explanatory tool for the *condition assessment* addressed to land managers. This has been incorporated into the guide to help land managers assess the condition of their peatland, which is the starting point for informing any changes in management or restoration required to improve condition and to leverage SNH funding (the condition assessment support tool is available here http://www.see.leeds.ac.uk/peatland-modules/?type=assess)
- *Live feedback forms* for gathering the public and land managers’ views and preferences regarding peatland restoration as part of the Peatland ACTION (available online at the end of the learning module and the condition assessment tool).

Furthermore, the framework developed here is currently being used by SNH as an information base to substantiate its investment in peatland restoration. At the moment of writing the Scottish Government has just released a draft Climate Change Plan which includes large peatland restoration targets (including the leverage of public funds) and promotes the use of communication and awareness raising tools. Personal communication with representatives of the Scottish Government and SNH indicate that the work presented here played a relevant role in influencing those decisions. Moreover, the SNH representative “[…] can see the value […] extending beyond Scotland as all governments are realising the need to assess non market benefits [of peatland restoration]” (Scotland Peatland Action’s coordinator letter of support to the research team).

Finally, we were invited to present the tool at a specific session on public perceptions at the International Union for Conservation of Nature’s (IUCN) conference ‘Creating a Legacy for Peatlands’ held in Shrewsbury (UK) in November 2016. As a follow up, the present contribution is made available to inform the forthcoming Commission of Inquiry on Peatlands and roll out IUCN’s UK Peatlands Programme communication Strategy planned for 2017 (Programme Communications Manager, personal communication). After presentation in the conference a number of peatland restoration practitioners expressed their interest in using the communication tools (the Wild Trust Wales and the Flow Country Peatland Partnership are just two examples of practitioners who expressed their interest). To satisfy those requests, the tool has been made freely available

## Conclusions

Ecosystems degradation represents one of the major global challenges at the present time, threating people’s livelihoods and well-being worldwide. Ecosystem restoration seems therefore no longer an option, but an imperative. However, understanding how ecosystem restoration works and can work is complex from whatever point is looked at. It is complex from the perspective of ecosystems’ natural functions and processes and from the perspective of restoration impacts and effectiveness. Restoration outcomes depend on the effects of climate change and anthropogenic pressures, with varying effects depending on baseline conditions, current status of degradation, location, restoration extent, etc. Restoration challenges are such that dialogue has begun on the need to re-shape restoration as a science and its contribution to restoration practice.

As part of that reshaping, it is becoming clearer that restoration science and practice needs to be coupled with socio-economic research and public engagement, so that socio-economic drivers and impacts of restoration can be incorporated into decision-making and public support can be gained for restoration on the ground. This unescapably requires conveying complex information in a way that is accessible for a wider public. How to do so in such a way that the representation of ecosystems remains scientifically rigorous is a critical challenge.

In this paper, we have taken up this challenge, using peatland as a paradigmatically complex ecosystem and Scotland as a case study to develop a transdisciplinary process to articulate a description of the processes and outcomes of peatland restoration. The ultimate aim is not an ontological simplification of the problem, but the development of tools that can support policy making decisions aimed at reversing the current degradation trend. In this respect, it is promising that the process resulted in the creation of materials that are now being used not only for communication with the public but also in restoration practice and other research contexts.

We believe that the four challenges identified in this paper (i.e.: (1) how to represent restoration outcomes; (2) how to establish a restoration reference; (3) how to cope with (varying) restoration time-lags and (4) how to define spatial units for restoration for which our process has provided evidence of usefulness), are relevant to ecosystem restoration generally beyond peatlands and Scotland. Similarly, both the overall transdisciplinary process as well as the specific mechanisms that we put in place (e.g. the photographic exercise) can be useful for other ecosystems and alternative approaches to ecosystem degradation (e.g. in addressing the economic, cultural and land-use drivers leading to degradation rather than restoration).

It should be noted, however, that this does not imply that the specific level at which we engaged with stakeholders in this research is the most appropriate in the *actual practice* of ecosystem restoration. As explained by [14], approaches to stakeholder participation have evolved from simply awareness raising [63], incorporating local perspectives in data collection and planning and developing techniques that recognised local knowledge [64, 65], to the use of participation as a norm in the sustainable development agenda of the 1990s [14]. After a phase of critique and disenchantment over its limitations, e.g. [66], there is now a focus on the identification of key features of best-practice to overcome failings from the past. A consensus seems to have emerged on the need to replace the ‘‘tool-kit” approach to participation, which emphasises selecting the relevant tools for the job, with an approach that views participation as a process [14]. Hence, there is no ‘right’ recipe for engaging with the public, but the process has to be underpinned by the appropriate ‘principles’ so that the public can fairly and effectively contribute. Public and stakeholder engagement can hence take different forms and needs to be specifically designed for the different contexts and purposes. What can undoubtedly be ascertained is that any such process will necessarily require a certain degree of simplification of the ecosystem’s complexity so that the public can understand and engage with it. This is what this paper has addressed.

In that respect, we believe that the most promising conclusion of the process that we put in place is of an epistemological nature. While ecosystem services-based approaches have certainly enhanced the integration of academic disciplines as well as non-specialist knowledge, interdisciplinary (and even transdisciplinary), processes attempting to put them into practice have so far tended to follow only one direction: i.e. let us first understand the ecosystems and the services they provide, describe them, categorize them, quantify them, and simplify them so that social sciences, the public and policy makers can understand them and operate with respect to them (i.e. from the biophysical underpinning *to* the public). Our process included mechanisms operating the other way round. The clearest case is that of the definition of spatial units for peatland restoration, where we tested the possibility of inverting this convention and started from what was pertinent and understandable for the public then worked our way back to relevant ecological parameters. Similarly, our photographic exercise to come up with the peatland categories also represents this ‘reverse’ approach.

We propose that it is the mix of approaches and epistemological directions (including also *from* the public to the biophysical parameters) what will make a definitive contribution to restoration practice.

## Supporting information

S1 Dataset Restoration complexity public survey raw data. (XLSX)
